# ‘Y Compartimos. . .’: the collective creation of performed fiction in practice

**DOI:** 10.1177/14744740241227442

**Published:** 2024-01-28

**Authors:** Joanna Kocsis

**Affiliations:** Newcastle University, UK

**Keywords:** arts-based research, Cuba, participatory research, performance, youth

## Abstract

This essay combines text and images in the style of a graphic novel to animate the lively and dynamic processes of a qualitative research approach that I call the collective creation of performed fiction. This is a form of projective storytelling in which participants draw on their own experiences to create and perform composite stories. Using fiction helps them avoid revealing sensitive details of their personal lives. The examples shared here are drawn from a long-term engagement with a group of youth in Old Havana, Cuba, where historic geopolitical tensions and emergent economic crises are interrupting the imagined futures of the young. This brief contribution documents key differences between three creative mediums used in this work (street theatre, film and animation), and addresses their varied capacities to mitigate the risks of self-disclosure.

As social science researchers, our job is to ask people to talk about their lives. Qualitative research often requires people who live in difficult or precarious contexts to disclose information about painful or traumatic experiences. There is a politics to such disclosure. Our research practices often probe for candid responses about socially, politically or personally sensitive topics, and there are times when disclosure may increase the risks faced by participants, especially for people who are vulnerable. These concerns are often heightened when working with children or youth.^1^ In discussions of research ethics, protection often hinges on confidentiality and ensuring participants’ anonymity. Confidentiality protects participants from harm, respects their right to privacy, and encourages candid responses.^2^ Increasing concerns about agency are pushing social scientists to innovate alternative ways to engage historically excluded groups, including young people, in knowledge production. How might we adapt our research practices to create space for young people to share the complex details of their lives while balancing concerns of protection? This article adds to the ways that geographers are expanding their methodological tool kits to include a range of creative practices.^3^ I argue that such practices can (not unproblematically) create new ways to mitigate the risks of disclosure.

In 2016, I began working with a group of youth in Cuba. The focus of this long-term participatory project has been to explore and understand the experiences of teenagers living Old Havana. In this article, I discuss our use of street theatre, film and animation. The value of creativity and performance in geographical work is well established and scholars note its potential for communicating sensitive topics,^4^ creating spaces for political resistance,^5^ collective healing,^6^ public debate,^7^ for staging alternative subjectivities^8,9^ and to explore, imagine and rehearse collective action.^10^ When used as a research method, however, creative methods can complicate questions of anonymity; in this case, by involving participants directly in public-facing performances and research outputs. To counter this, our project created fictional composite stories (based on real experiences) to maintain confidentiality, making it impossible to determine whose experiences informed our creative work. Shielding behind fiction provided the opportunity for these Cuban teens to transgress taboos and to share their experiences of sensitive topics such as illegal livelihoods, violence, sexuality and illegal migration. I present this collective work in the form of a graphic novel.


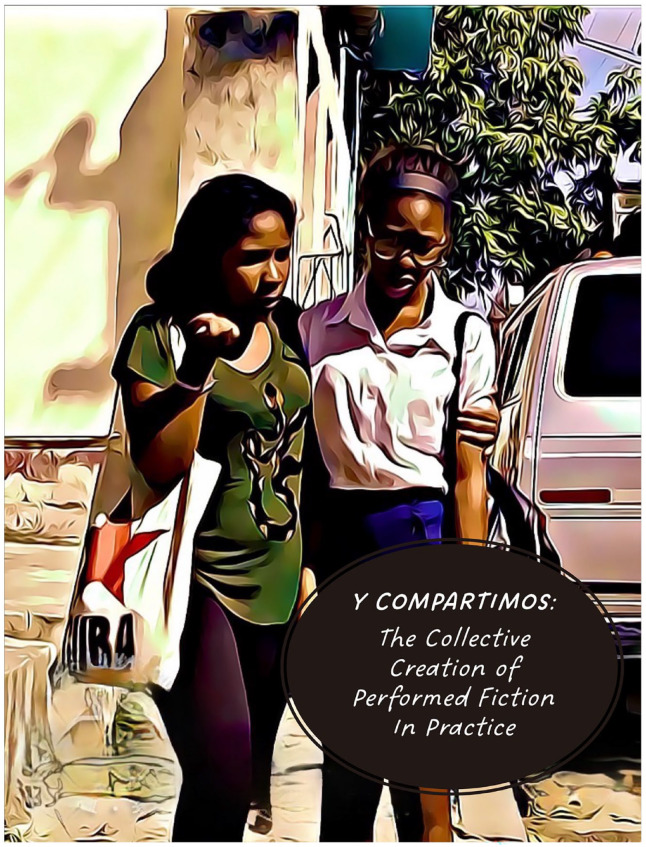



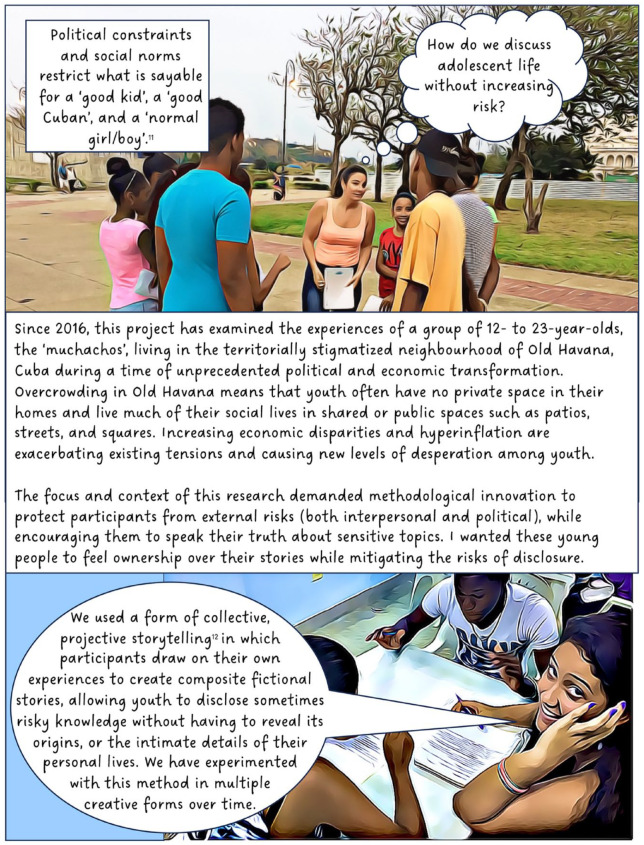



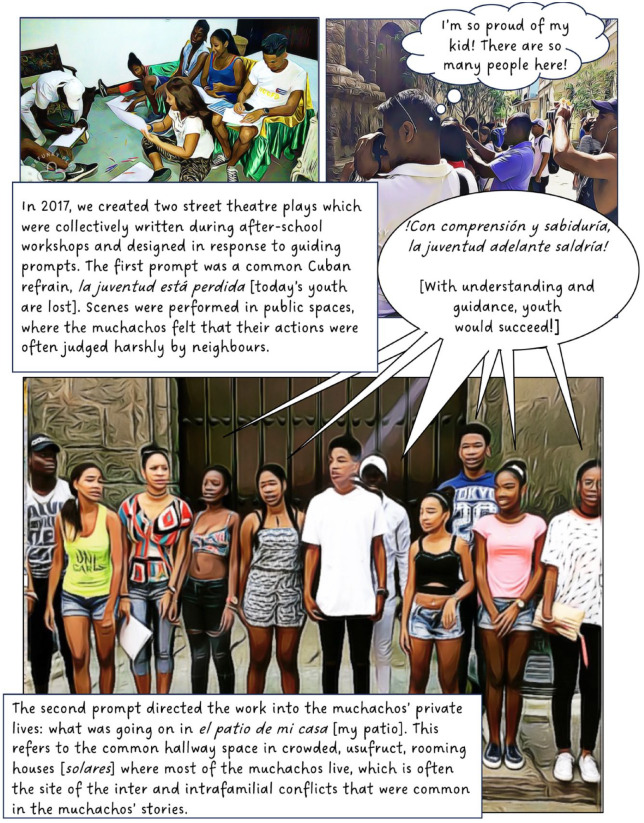



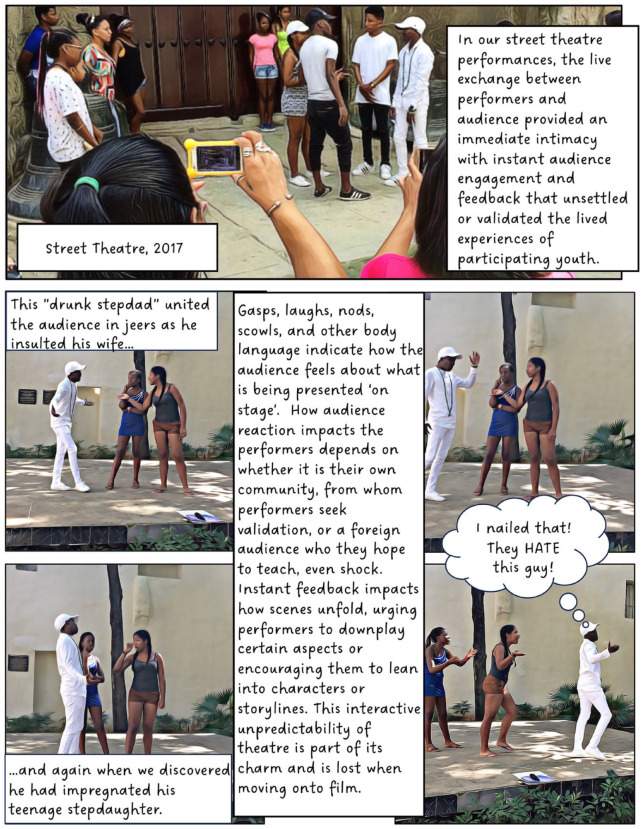



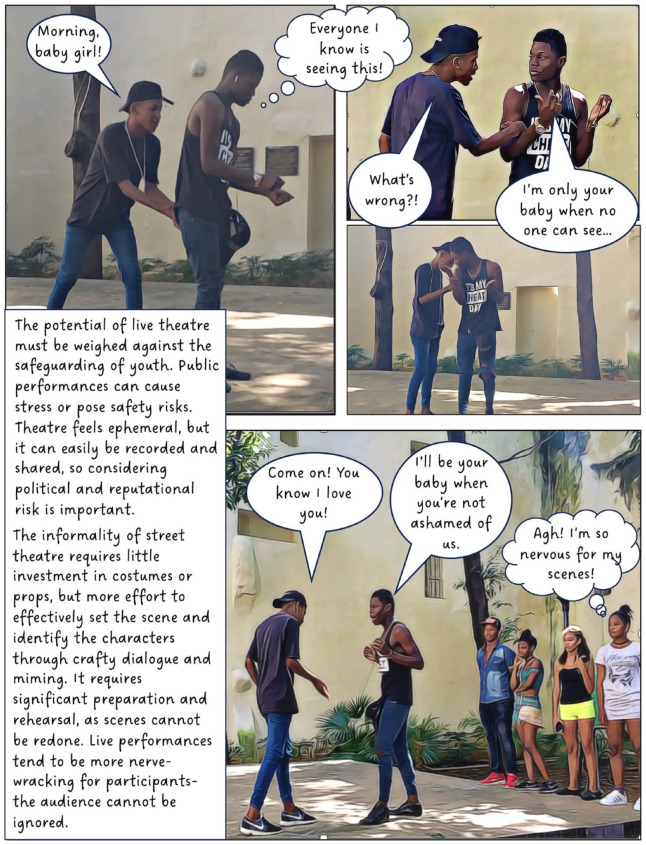



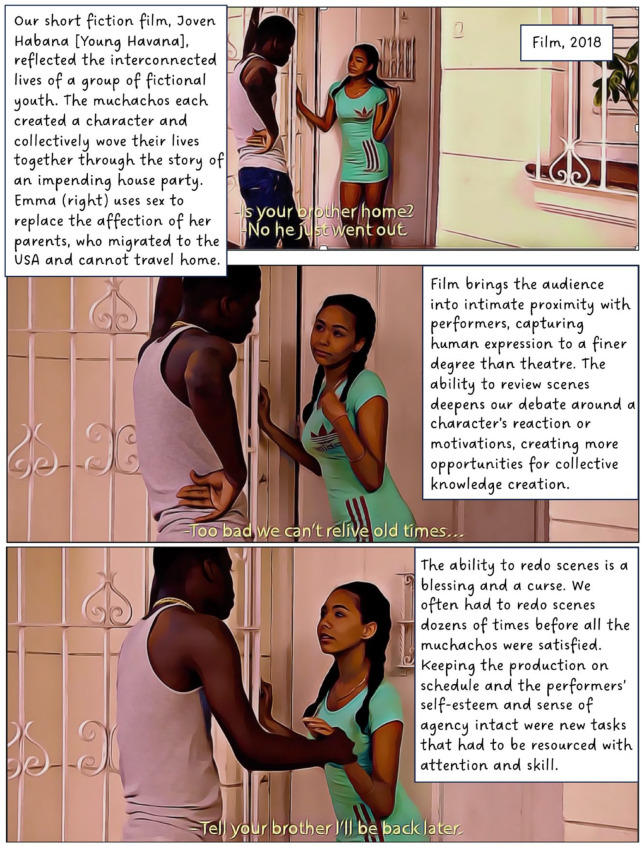



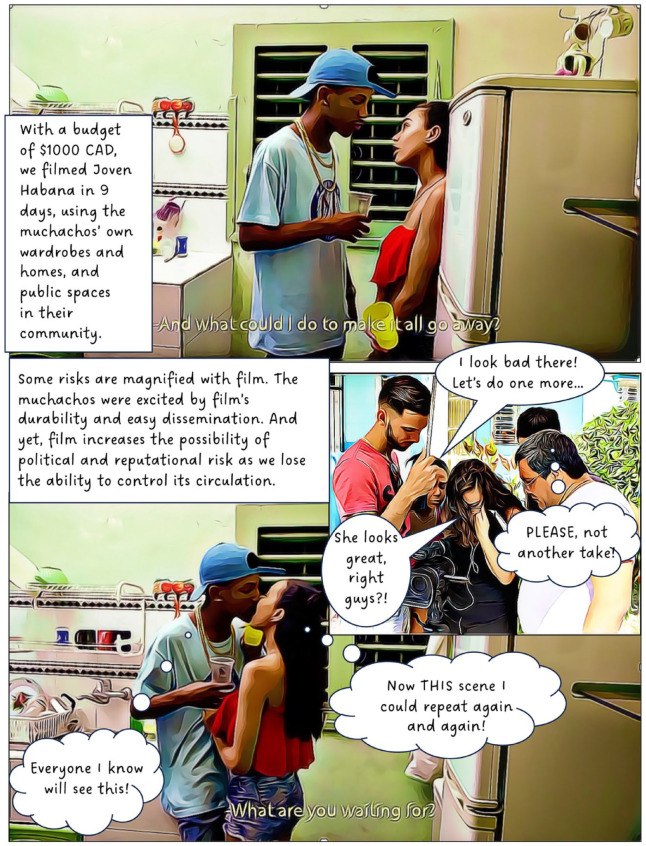



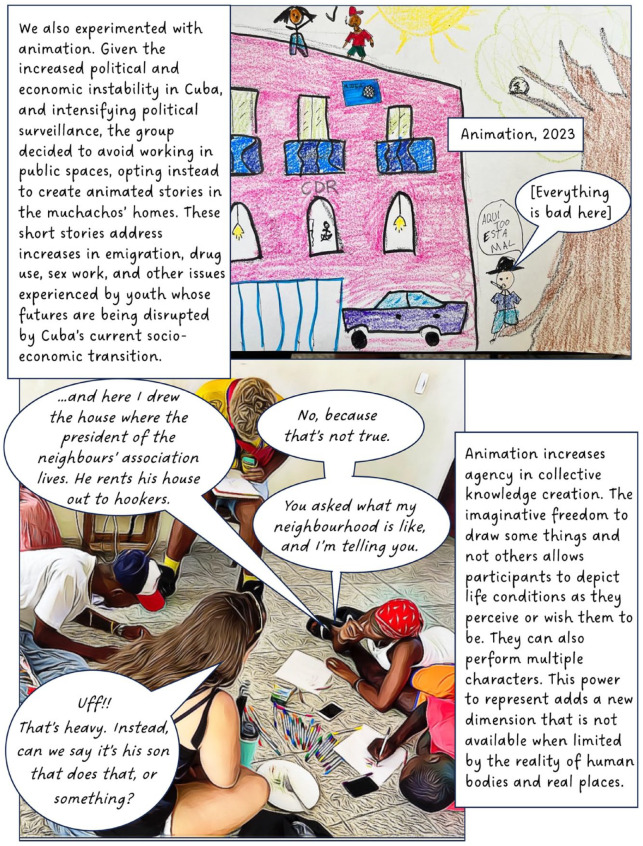



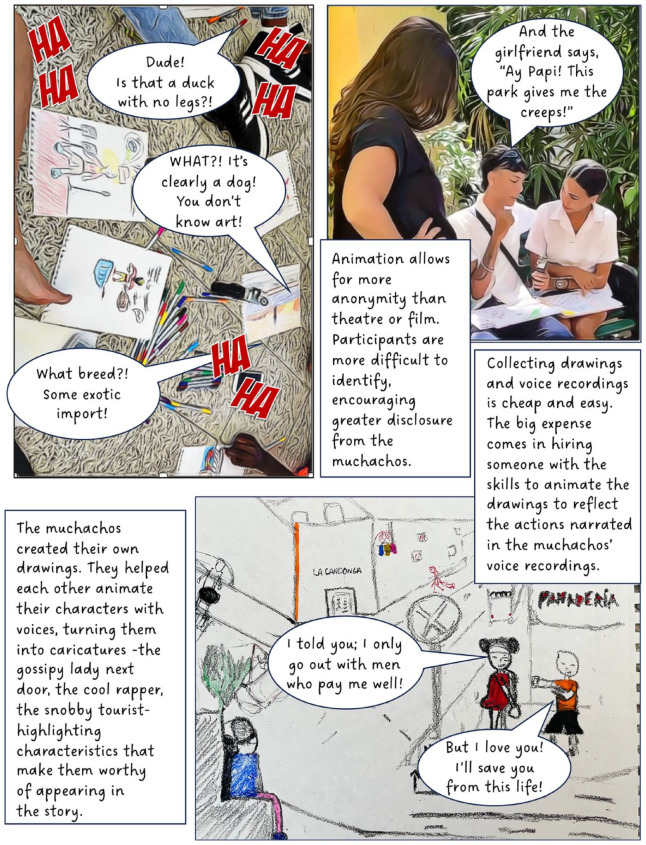


## Concluding thoughts

Through this project, we learned how different creative mediums (street theatre, film, animation) can work in different ways to offer participants space and means to disclose experiences considered taboo or risky. Our creative work opened spaces for the muchachos to share intimate details about their everyday lives and experiences on a range of issues. Yet, such disclosure is never uncomplicated, and there are risks for youth speaking out in Old Havana. To ensure their relative ‘protection’, the group created composite fictional stories which formed the basis of our public-facing creative work and performances. These composite stories (based on real experiences) allowed us to retain politically charged details without exposing youth to additional personal risk. Ensuring anonymity has become even more significant since 2021, in the wake of increasing political resistance in response to the rapid socio-political and economic transformation in their community and country. The collective creation of performed fiction has proven a valuable tool for balancing agency and risk. Understanding the protections provided by different mediums supports geographers experimenting with creative methods to better conceive of ways that both empower and take care of their participants.

## Notes

1. V.Morrow and M.Richards, ‘The Ethics of Social Research with Children: An Overview’, *Children & Society*, 10(2), 1996, pp. 90–105.

2. B.Baez, ‘Confidentiality in Qualitative Research: Reflections on Secrets, Power and Agency’, *Qualitative Research*, 2(1), 2002, pp. 35–58.

3. H.Hawkins, ‘Geography’s Creative (Re) Turn: Toward a Critical Framework’, *Progress in Human Geography*, 43(6), 2019, pp. 963–1152.

4. D.Conquergood, ‘Performance Studies: Interventions and Radical Research’, *TDR*, 46(2), 2002, pp. 145–56.

5. G.Pratt and C.Johnston, ‘Turning Theatre into Law, and Other Spaces of Politics’, *cultural geographies*, 14(1), 2007, pp. 92–113.

6. J.B.Miller, ‘The Transformative and Healing Power of Theatre of Witness’, *Canadian Journal for the Study of Adult Education*, 30(2), 2018, p. 47.

7. G.Pratt and C.Johnston, ‘Staging Testimony in Nanay’, *Geographical Review*, 103(2), 2013, pp. 288–303.

8. C.Johnston and D.Bajrange, ‘Street Theatre as Democratic Politics in Ahmedabad’, *Antipode*, 46(2), 2014, pp. 455–76.

9. H.Hawkins, ‘Creative Geographic Methods: Knowing, Representing, Intervening. On Composing Place and Page’, *cultural geographies*, 22(2), 2015, pp. 247–68.

10. A.Cole, L.Fontana, M.Hirzel, C.Johnston and A.Miramonti, ‘On Burning Ground: Theatre of the Oppressed and Ecological Crisis in Bolivia’, *cultural geographies*, 30(4), 2023, pp. 639–48.

11. J.Kocsis, ‘‘¡Eso No Se Dice’!: Exploring the Value of Communication Distortions in Participatory Planning’, *Planning Theory*, 22(3), 2023, pp. 270–91.

12. C.Kitzinger and D.Powell, ‘Engendering Infidelity: Essentialist and Social Constructionist Readings of a Story Completion Task’, *Feminism & Psychology*, 5(3), 1995, pp. 345–72.

